# Endoplasmic Reticulum Stress Activation in Alport Syndrome Varies Between Genotype and Cell Type

**DOI:** 10.3389/fgene.2020.00036

**Published:** 2020-02-10

**Authors:** Cong Wang, Shenghui Liang, Shijia Xing, Ke Xu, Huijie Xiao, Haiyue Deng, Xiaoyuan Wang, Liangyi Chen, Jie Ding, Fang Wang

**Affiliations:** ^1^ Department of Pediatrics, Peking University First Hospital, Beijing, China; ^2^ State Key Laboratory of Membrane Biology, Beijing Key Laboratory of Cardiometabolic Molecular Medicine, Institute of Molecular Medicine, Peking University, Beijing, China

**Keywords:** *COL4A5* mutation, Alport syndrome, induced pluripotent stem cell, fibroblast, endoplasmic reticulum stress

## Abstract

Alport syndrome is a hereditary progressive chronic kidney disease caused by mutations in type IV collagen genes *COL4A3/4/5*. X-linked Alport syndrome (XLAS) is caused by mutations in the *COL4A5* gene and is the most common form of Alport syndrome. A strong correlation between the type of *COL4A5* mutation and the age developing end-stage renal disease in male patients has been found. Mutation to the α (IV) chain causes retention of the protein to the endoplasmic reticulum lumen, which causes endoplasmic reticulum stress (ERS) and subsequent exertion of deleterious intracellular effects through the activation of ERS. The exact time point that mutant type IV collagen α chain exerts its deleterious effects remains elusive. In this study, we explored the relationship between the *COL4A5* genotype and cell type in ERS activation. We obtained skin fibroblasts from Alport syndrome patients with different *COL4A5* mutation categories [i.e., a missense mutation (c.4298G > T, p.Gly1433Val) in exon 47, a splicing mutation (c.1949–1G > A) in intron 25 and an insertion (c.573_c.574insG, p. Pro193Alafs*23) in exon 10], and then reprogrammed these fibroblasts into induced pluripotent stem cells (iPSCs). Interestingly, no significant dysregulation of ERS pathway markers was observed for the three *COL4A5* mutant iPSCs; however, significant activation of ERS in *COL4A5* mutant fibroblasts was observed. In addition, we found that the activation levels of some ERS markers in fibroblasts varied among the three *COL4A5* mutation types. Mutant *COL4A5* proteins were demonstrated to have different effects on cells at different stages of ontogenesis, providing a theoretical basis for choosing the timing of intervention. The observed differences in activation of ERS by the *COL4A5* mutant fibroblasts may contribute to the intracellular molecular mechanisms that describe the correlation between genotype and clinical features in XLAS.

## Introduction

Alport syndrome (OMIM, X-LINKED, TS1, # 301050; AUTOSOMAL RECESSIVE, TS2, # 203780; AUTOSOMAL DOMINANT, TS1, # 104200) is caused by mutations in *COL4A3/4/5* genes, which encode type IV collagen α3/4/5 chains (α3 chain, UniProt Q01955; α4 chain, P53420; α5 chain, P29400). Alport syndrome is a progressive kidney disease that gives rise to hematuria during the early stages of the disorder and then proteinuria and the progressive decline of renal function, which ultimately leads to renal failure. Patients with Alport syndrome usually develop end-stage renal disease (ESRD) when they are young (Jais et al., 2000; Kruegel et al., 2013). Clinical diagnosis of this disorder can be made when one of the following four criteria are met: a) abnormal staining of the α5 (IV) chain in the epidermal basement membrane (EBM); b) abnormal staining of the α (IV) chains in the glomerular basement membrane (GBM); c) mutations in the *COL4A3/4/5* genes; and d) ultrastructural changes in the GBM typical of Alport syndrome (Wang et al., 2012; Savige et al., 2013). Approximately 80% of Alport syndrome patients are X-linked (XLAS) (OMIM # 301050) and because of mutations in the *COL4A5* gene (located at position Xq22.3, OMIM 301050, RefSeq Z37153, HGNC 2207; NCBI reference sequence NM_000495.4) (Jais et al., 2000).

Type IV collagen has six α chains and each α chain has a very similar primary structure, which is characterized by a N-terminal 7S domain, a long central triple-helical collagenous domain of Gly-X-Y repeats and a C-terminal non-collagenous (NC) domain (Hudson et al., 2003; Mao et al., 2015). The type IV collagen α3, α4, and α5 network predominates in the mature GBM structure and is derived exclusively from podocytes (Abrahamson et al., 2009; Kruegel et al., 2013). Physiologically, the type IV collagen α3, α4, and α5 chains are translated, undergo post-translational modification and assemble in the endoplasmic reticulum (ER) to form a triple-helix heterotrimer. This heterotrimer is distributed by the Golgi apparatus and then secreted into the GBM (Khoshnoodi et al., 2008; Chioran et al., 2017). The ER is a fundamental organelle for protein synthesis and for maintaining protein homeostasis. Mutant proteins, such as the type IV collagen α5 chain, can disrupt normal protein synthesis (Kobayashi et al., 2008), cause accumulation of unfolded and misfolded proteins in the ER and result in the activation of ER stress (ERS), which is mediated by the unfolded protein response (UPR) (Wang et al., 2017; Park et al., 2019). The UPR can restore ER function and may be protective during early stages of ERS; however, sustained ERS caused by a mutant protein can be cytotoxic (Cybulsky, 2017; Park et al., 2019).

Mutations in *COL4A3/4/5* genes occur during the early stage of the fertilized ovum and persist throughout life in Alport syndrome patients. A mutant type IV collagen α3 chain is retained in the ER lumen and causes ERS mediated by UPR, which exerts deleterious intracellular effects (Pieri et al., 2014). The exact period that abnormal type IV collagen exerts its deleterious effects remains elusive.

In 2006, induced pluripotent stem cells (iPSCs) were established by direct reprogramming of differentiated somatic cells. This technology demonstrated that mature somatic cells can be reprogrammed into their initial state (Takahashi and Yamanaka, 2006), and iPSC is an embryonic stem cell like pluripotent cell (Takahashi and Yamanaka, 2006; Yu et al., 2007). This technology enables the study of the effects of causative gene mutations on type IV collagen at the iPSC level.

In this study, dermal fibroblasts from Alport syndrome patients with different *COL4A5* gene mutations were reprogrammed into iPSCs. We explored ERS in these iPSCs and somatic cells to characterize the difference in UPR activation of undifferentiated and differentiated cells to provide a theoretical basis for intervention time.

## Materials and Methods

### Patients

The Ethics Committee of the Peking University First Hospital approved this study and written informed consent to participate and written informed consent to publish were obtained from the parents of the participants in the study. Three boys with different *COL4A5* gene mutations were included in this study. Patient 1 was a 3-year-old boy with microscopic hematuria and microalbuminuria for 1 year. Hematuria and proteinuria were detected in his mother when she was pregnant with the boy. Negative and mosaic α5 (IV) chain immunostaining in the EBM were observed in this patient and his mother, respectively. A hemizygous missense mutation c.4298G > T (p.Gly1433Val) in the *COL4A5* gene was detected by genomic DNA analysis, and the same heterozygous mutation was traced to his mother. Patient 2 was a 12-year-old boy with microscopic hematuria, nephrotic-level proteinuria and renal dysfunction. Negative immunostaining of the α5 (IV) chain in the EBM was observed in this patient. A hemizygous splicing mutation c.1949–1G > A in the *COL4A5* gene was detected in his blood sample. Patient 3 was a 3-year-old boy with microscopic hematuria. He had a maternal family history of hematuria and ESRD. Negative immunostaining of the α5 (IV) chain in the EBM was observed. Genomic DNA analysis revealed a hemizygous frameshift mutation c.573_574insG (p.Gly192Glyfs*24) in the *COL4A5* gene in this patient, and his mother harbored the same heterozygous mutation.

Missense mutation c.4298G > T (p.Gly1433Val) in exon 47 of the *COL4A5* gene disrupts the Gly-X-Y repeats in the collagenous domain of the collagen IV α5 chain. Splicing mutation c.1949-1G > A in intron 25 and insertion c.573_c.574 insG in exon 10 were predicted to produce a truncated α5 (IV) chain, and the latter mutant protein lacked the NC domain.

### Cell Culturing

Skin fibroblasts were cultured according to a method described previously (Wang et al., 2005). Dermal fibroblasts were maintained in high glucose DMEM (Invitrogen, Cat NO. 10569010) with 10% FBS (Gibco, Cat NO.10099141), 1% penicillin/streptomycin (Invitrogen, Cat NO.15140122) and 0.1 mM non-essential amino acids (Gibco, Cat NO. 11140050) at 37°C in a 5% CO_2_ incubator. Human iPSCs were cultured on Matrigel (Corning, Cat NO. 354277) coated dishes in mTeSR medium (STEMCELL Technologies, Cat NO. 85850) at 37°C and 5% CO_2_.

### Reprogramming of Fibroblasts

Dermal fibroblasts from Alport syndrome patients with different *COL4A5* gene mutations and one normal (control) fibroblast (Life Technologies, Cat NO.C-004-5C) were used to reprogram iPSCs. After reaching approximately 75%–90% confluence, 1 × 10^5^ fibroblasts were transfected with five reprogramming plasmids carrying Oct4, Sox2, Kfl4, L-Myc, Lin28, and mp53DD to knockdown of p53 to improve reprogramming efficiencies using the Neon Transfection System (Gibco, Cat NO. MPK5000). After electroporation, the fibroblasts were maintained in DMEM/F-12 with HEPES (Invitrogen, Cat NO. 11330032) supplemented with 1× N-2 supplement (Invitrogen, Cat NO. 17502-048), 1× B-27 supplement (Invitrogen, Cat NO. 17504-044), 0.1 mM MEM non-essential amino acids (Invitrogen, Cat NO. 11140050), 2 mM GlutaMAX-I (Invitrogen, Cat NO. 35050-061), 100 μM β-mercaptoethanol (Invitrogen, Cat NO. 21985023), and 100 ng/mL basic FGF (Peprotech, Cat NO. AF-100-18B). Basic FGF was added when the medium was used. The electroporation parameters were as follows: pulse voltage, 1650 V; pulse width, 10 ms; and pulse number, 3. The medium was replaced every second day and the medium was switched to mTeSR medium at day 15 of post-transfection. iPSC colonies began to appear at this time point. At 21 days transfection, iPSC colonies of appropriate size were transferred to matrigel-coated 48-well plates. The iPSC colonies were passaged onto Matrigel-coated 48-well plates in mTeSR medium for expansion once they had increased in size.

### Karyotype Analysis

After harvesting the iPSCs, the cells were treated in a medium with 0.2 g/ml colchicine for 20 min. After centrifugation, the iPSCs were treated with a low osmotic pressure fluid for 15–17 min. The cells were fixed with 4% paraformaldehyde. After fixation, the resuspended cells were dropped onto microslides and then dried at 90°C for 2 h. The cells on the microslides were stained with Giemsa and observed by a Leica Automatic slide scanning system GSL-120.

### Alkaline Phosphatase of iPSCs

To detect alkaline phosphatase activity, iPSCs were fixed with 4% paraformaldehyde at room temperature for 1 min. After washing with PBS, iPSCs were incubated with BCIP/NBT alkaline phosphatase color development solution (Beyotime, Cat NO. C3206) in the dark at room temperature for 30 min. After removing the BCIP/NBT alkaline phosphatase color development solution, the reactions were terminated by washing with distilled water. Images were taken using a microscope (Olympus CKX41, Tokyo, Japan).

### Immunocytochemistry for Pluripotency Markers

Immunofluorescence staining was performed to detect pluripotency markers. iPSCs of Alport syndrome patients and the healthy control were fixed with 4% paraformaldehyde, blocked with PBS containing 0.3% Triton X-100, and 10% BSA for 1 h. Primary antibodies against Nanog (SANTA CRUZ, Cat NO. sc-293121) and Oct-3/4 (SANTA CRUZ, Cat NO. sc-5279) in PBS containing 0.3% Triton X-100 and 1% BSA were then incubated with the fixed cells at 4°C overnight. After washing three times with PBS containing 0.1% Triton X-100 for 30 min, cells were incubated with secondary antibodies conjugated with Alexa Fluor 561 (Thermo Fisher Scientific, Cat NO. A-11062) at 4°C overnight. The iPSCs were stained with DAPI. Fluorescence images were taken using a confocal fluorescence microscope (Olympus IX81, Tokyo, Japan).

### Trilineage Differentiation of iPSCs *In Vitro*

For mesoderm lineage and endoderm lineage differentiation, the iPSC clones were transferred onto Matrigel coated 12-well plates in mTeSR medium supplemented with 10 µmol/L Y-27632 (Abcam, Cat NO. ab120129). The following day the culture medium was changed to mesoderm or endoderm single-cell plating medium (STEMCELL Technologies, Cat NO. 05232 and Cat NO.05233, respectively), and the differentiation media were replaced every day. At day 5 the cells were fixed with 4% paraformaldehyde for analysis of mesoderm and endoderm markers Brachyury (Biotechne, Cat NO. MAB20851) and Sox17 (R&D Systems, Cat NO. AF1924) by immunofluorescence staining, respectively.

For ectoderm lineage differentiation, the iPSC clones were transferred onto Matrigel coated 12-well plates in ectoderm single-cell plating medium (STEMCELL Technologies, Cat NO. 05231) supplemented with 10 µmol/L Y-27632. The following day the culture medium was changed to the medium without Y-27632, and the differentiation medium was replaced every day. At day 7, the cells were fixed with 4% paraformaldehyde for analysis of the ectoderm marker, Otx2 (Biotechne, Cat NO. AF1979). The trilineage specific markers were analyzed by immunofluorescence staining.

### *COL4A5* Gene Analysis

Genomic DNA of Alport syndrome fibroblasts and iPSCs were extracted using the Blood and Tissue Kit (Qiagen, Cat NO. A1120). Exons 47, 26, and 10 of the *COL4A5* gene were amplified by PCR. Three pairs of primers were used. *COL4A5* Exon 47 forward primer: 5'-TGTTTTGTCAATATCCATAAGAGTGG-3'; *COL4A5* Exon 47 reverse primer: 5'-GGCCAAGGCTACTCTAGAACC-3'; *COL4A5* Exon 26 forward primer: 5'-GGGTGGATCATCCTTATTCG-3'; *COL4A5* Exon 26 reverse primer: 5'-CAGCAAGCCAACATCACG-3'; *COL4A5* Exon 10 forward primer: 5'-AGAGCAGAATTCCAATGACG-3'; and *COL4A5* Exon 10 reverse primer: 5'-TTATGAAGCCCTGCTTTTGC-3'. The PCR products were subsequently sequenced with an ABI 3730XL Genetic Analyzer.

### mRNA Expression of Mutant *COL4A5*

Initially, commercial antibodies recognizing the N- and C-termini of α5 (IV) were used to analyze the expression of the type IV collagen α5 chain in skin fibroblasts and iPSCs. However, both antibodies did not specifically detect α5(IV). Therefore, mRNA expression of *COL4A5* was examined.

Total RNA was extracted from the corresponding skin fibroblasts and iPSCs using the Trizol reagent (Thermo Fisher Scientific, Cat NO. 15596018), and cDNA synthesis was performed using reverse transcriptase (Thermo Fisher Scientific, Cat NO. K1622). cDNA was used with specific primers containing the mutation site, as described previously (Wang et al., 2005). The forward primer used in the missense mutation was 5'-GGATTCCCAGGCATGAAAGG-3' and the reverse primer was 5'-CCTTTAGGGGTTGCATGCTC-3'. The forward primer used in the splicing mutation was 5'- AGGAGAACAAGGAGTGAAAGGT-3' and the reverse primer was 5'-CTATTGGCCCAGGAATCCC-3'. The forward primer used in the frameshift mutation was 5'-ATGGAACCAAGGGAGAACGT-3' and the reverse primer was 5'-GCCCTTTTCACCATCCCTTC-3'. The PCR products were examined by 3% agarose gel electrophoresis and then sequenced as described above. Fibroblasts from the 5-year-old boy who did not suffer Alport syndrome were used as the normal control.

### Western Blotting

The cells were lysed in RIPA buffer (PPLYCEN, Cat NO. 1053) and a protease inhibitor cocktail (Roche, Cat NO. 04693159001) was added. Twenty micrograms of protein were loaded onto SDS-PAGE gels to separate proteins, and separated proteins were transferred to nitrocellulose filter membranes (Amersham, Cat NO. 10600002). After blocking in 5% skimmed milk, the membranes were incubated overnight at 4°C with the primary antibodies. On the second day, after washing with PBS containing 1% Tween, the membranes were incubated with secondary antibodies conjugated with horseradish peroxidase at room temperature for 1 h. Proteins bound to the membrane were visualized by the detection system (GBOX-CHEMI-XT4, GENE, Hong Kong, China) using an ECL kit (Millipore, WBKLS0100). The densities of each band were normalized to β-tubulin. Primary antibodies for western blotting included activating transcription factor 6 (ATF-6, Proteintech, Cat NO. 24169-1-AP), phosphorylated inositol requiring enzyme 1α (p-IRE1α, Thermo Fisher Scientific, Cat NO. PA1-16927), phosphorylated protein kinase RNA like ER kinase (p-PERK, Affinity Biosciences, Cat NO. DF7576), binding Ig protein (Bip, Cell Signaling Technology, Cat NO. 3177), inositol-requiring enzyme 1α (IRE1α, Cell Signaling Technology, Cat NO. 3294), protein kinase RNA like ER kinase (PERK, Cell Signaling Technology, Cat NO. 5683) and β-tubulin (ZSGB-Bio, Cat NO. TA-10). For the detection of ERS activation in Alport syndrome fibroblasts using western blotting, the fibroblasts from the 5-year-old boy who did not suffer Alport syndrome were used as the normal control.

### Statistical Analysis

SPSS version 19.0 software (IBM SPSS, Chicago, IL, USA) was used for all statistical analyses. Results are expressed as means ± SEM. For comparisons involving two groups, the independent samples t-test was chosen. A *p* value less than or equal to 0.05 was considered statistically significant, and all experiments were performed at least three times independently.

## Results

### Generation of Alport Syndrome Specific iPSCs

**Figure 1:**
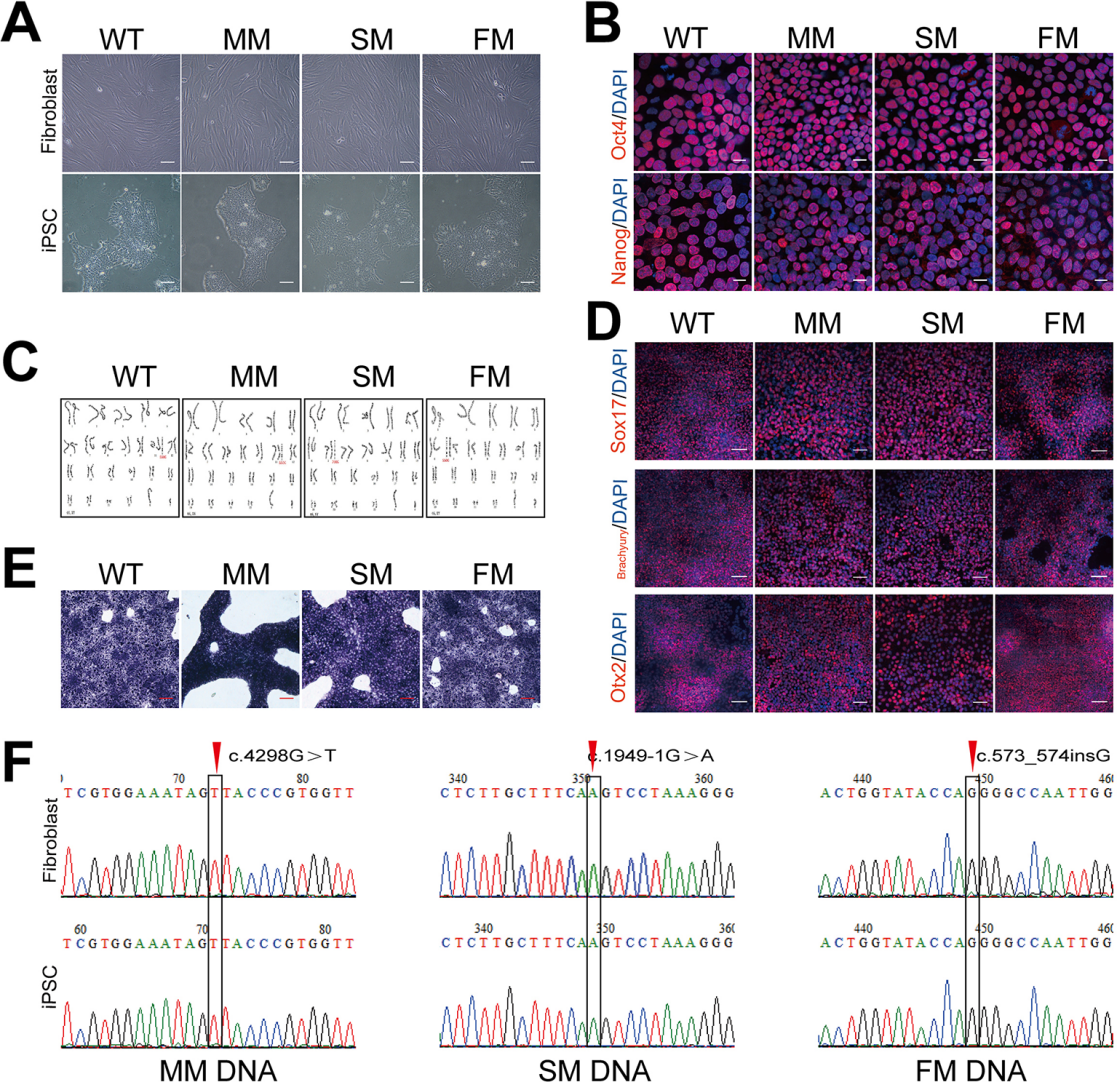
Generation of WT and Alport iPSCs. **(A)** The morphology of fibroblasts and fibroblast- derived iPSCs. Scale bar, 100 μm. **(B)** Immunofluorescence staining of pluripotency markers, Oct4 (red) and Nanog (red). Nuclei were stained with DAPI (blue). Scale bar, 20 μm. **(C)** Karyotyping analysis of control (WT) and Alport syndrome patient iPSCs. **(D)** *In vitro* immunofluorescence staining of Sox17 (endoderm) (red), Brachyury (mesoderm) (red), and Otx2 (ectoderm) (red) for the three germ layers of WT and Alport syndrome patient iPSCs. Nuclei were stained with DAPI (blue). **(E)** Alkaline phosphatase staining of WT and Alport syndrome patient iPSCs. Scale bar, 100 μm. **(F)** Confirmation of the mutations of fibroblasts and iPSCs in *COL4A5* missense mutation c.4298G > T, splicing mutation c.1949-1G > A and frameshift mutation c.573_574insG. The right one represents the direct sequencing of *COL4A5* splicing mutation cDNA from skin fibroblasts and iPSCs. The red arrow and black frame indicate the mutation sites. (MM, missense mutation; SM, splicing mutation; FM, frameshift mutation).

**Figure 2:**
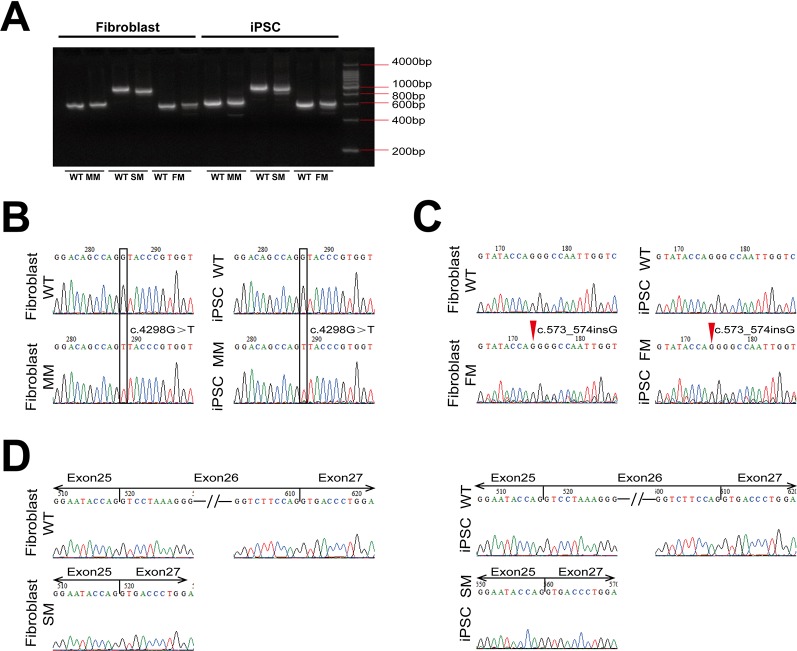
The expression of *COL4A5* variants in fibroblasts and iPSCs. **(A)** RT-PCR amplification was used with specific primers containing the mutation site of *COL4A5*. **(B–D)** *COL4A5* sequence variants in the missense mutation **(B)**, frameshift mutation **(C)**, and splicing mutation **(D)** fibroblasts and iPSCs identified by Sanger sequencing. (MM, missense mutation; SM, splicing mutation; FM, frameshift mutation).

We obtained human dermal fibroblasts from three patients with X-linked Alport syndrome (harboring missense, splicing and frameshift mutations in the *COL4A5* gene) and one healthy control (WT), and generated patient-specific and WT iPSCs. The fibroblasts were transfected with the pluripotency genes Oct4, Sox2, Klf4, L-Myc, and Lin28, as well as simultaneous knockdown of p53 to improve reprogramming efficiencies. The morphology of Alport syndrome fibroblasts and the generated iPSCs changed from a spindle-shaped, single cell layer to round or oval shaped cells and colonies grew formed cells (**Figure 1A**). The four generated iPSCs exhibited pluripotency markers Oct4 and Nanog (**Figure 1B**). The generated iPSCs developed into three germ layers *in vitro* (**Figure 1D**), maintained normal karyotypes (**Figure 1C**) and expressed alkaline phosphatase (**Figure 1E**). Hemizygous mutations of the *COL4A5* gene [(c.4298G > T, p.G1433V), (c.1949-1G > A, IVS25), and (c.573_c.574insG, p. G192Gfs*24)] in fibroblasts and iPSCs of Alport syndrome patients were confirmed by genomic PCR and Sanger sequencing (**Figure 1F**). In addition, direct sequencing of *COL4A5* cDNA from skin fibroblasts and iPSCs revealed that splicing mutation c.1949-1G > A arises from complete exon 26 skipping (r. 1949_2041del) (**Figure 2D**). Taken together, Alport syndrome-specific iPSCs were generated with normal pluripotency.

### mRNA Expression of Mutant *COL4A5* in Fibroblasts and iPSCs

As shown in **Figure 2A**, reverse transcriptase-PCR analysis confirmed expression of the mutant *COL4A5* transcripts in fibroblasts and iPSCs. The same missense mutation and insertion were confirmed at the cDNA level (**Figures 2B, C**), and the splicing mutation c.1949-1G > A resulted in aberrant splicing of mRNA, causing complete exon 26 skipping (r. 1949_2041del) (**Figure 2D**).

### ERS Regulation in Alport Syndrome Specific iPSCs

**Figure 3:**
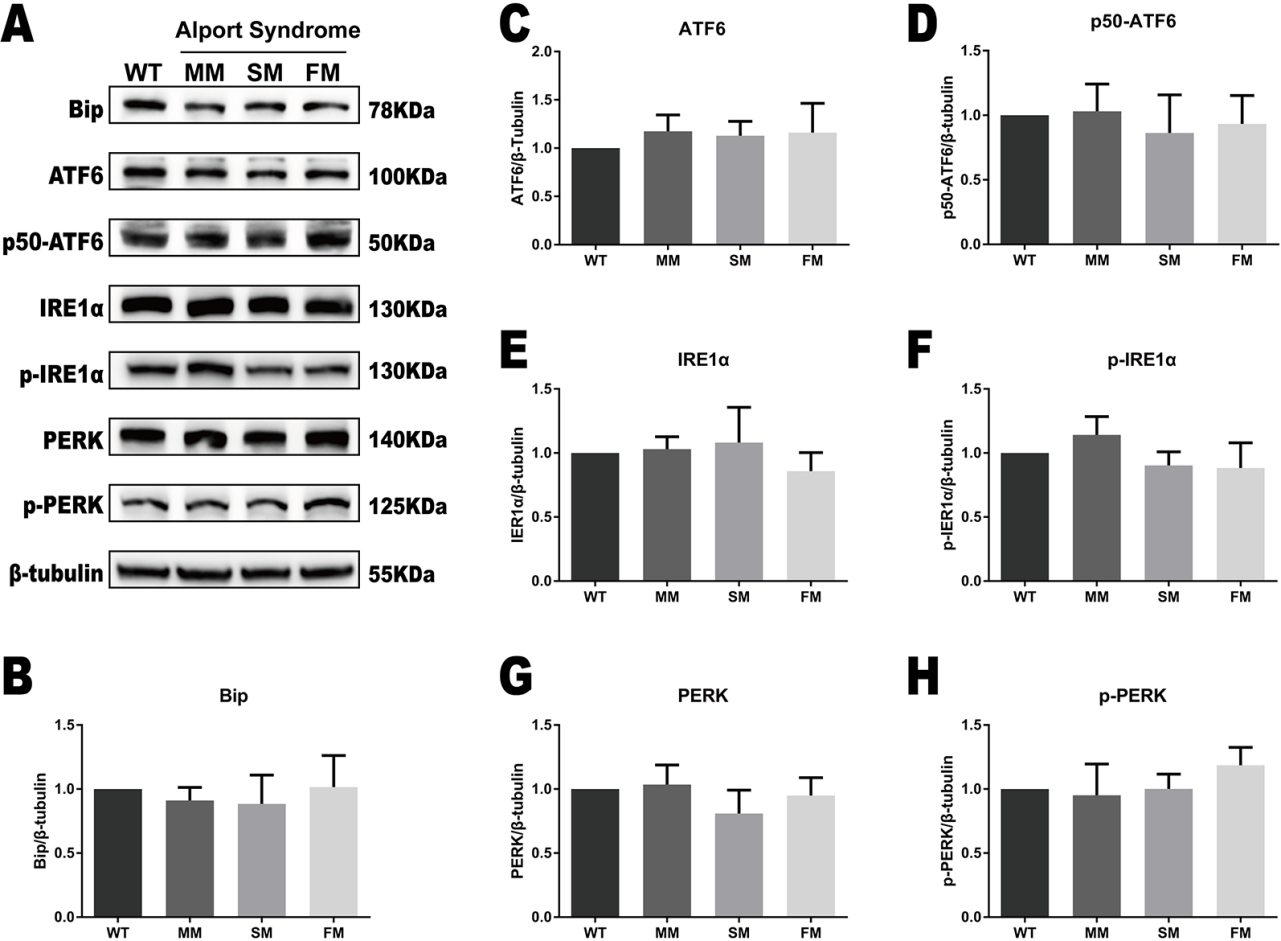
The UPR protein levels are similar in WT and *COL4A5* mutant iPSCs. **(A)** Protein expression of the UPR markers Bip, ATF6, p50-ATF6, p-IRE1α, IRE1α, p-PERK, and PERK was measured after reprogramming by western blotting. β-tubulin expression was used as an equal loading control. Representative blotting shows the protein levels of these proteins (**Supplementary Figure 1**). **(B–H)** The expression of Bip, ATF6, p50-ATF6, p-IRE1α, IRE1α, p-PERK, and PERK in WT iPSCs, *COL4A5* missense mutant iPSCs, *COL4A5* splicing iPSCs and *COL4A5* frameshifted mutant iPSCs were quantified by densitometric analysis. The protein levels of ER chaperone protein Bip, three ER transducers (ATF6, IRE1α, and PERK) and their activated forms (p50-ATF6, p-IRE1α, and p-PERK) were similar between the WT and mutant samples examined. Data are means ± SEM. *n* = 3, (WT, control; MM, missense mutation; SM, splicing mutation; FM, frameshift mutation).

We tested for protein expression of the ERS markers Bip, ATF6, IRE1α, and PERK, as well as their active forms in the WT and Alport syndrome-specific iPSCs. As showed in **Figure 3**, no significant change was observed in the expression levels of Bip, ATF6, IRE1α, and PERK, or their active forms, activated ATF6 (p50-ATF6) and the phosphorylated forms of IRE1α (p-IRE1α) and PERK (p-PERK).

### Verification of ERS Activation in Alport Syndrome Fibroblast Using Western Blotting

**Figure 4:**
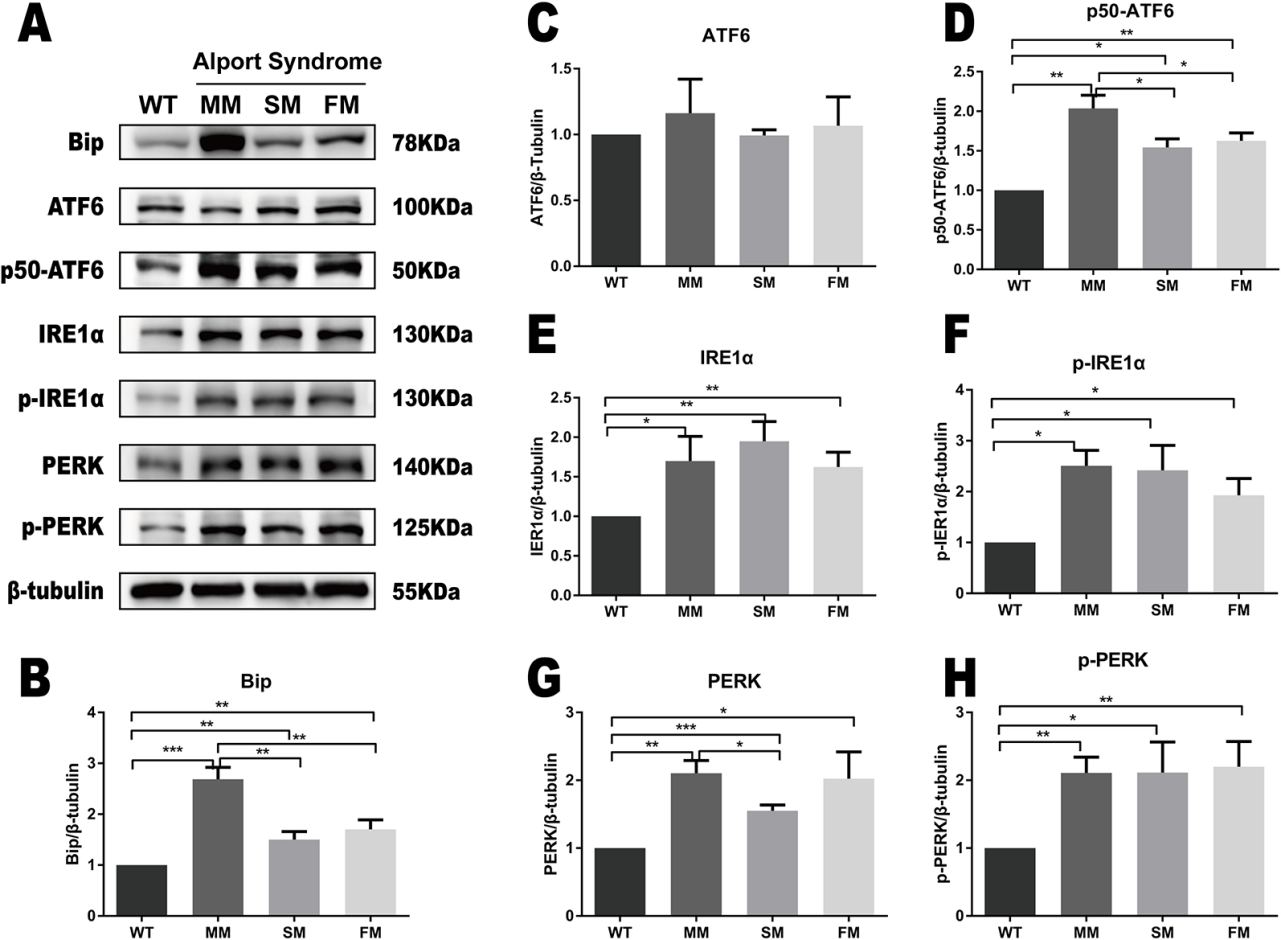
ERS pathway was activated in *COL4A5* mutant fibroblasts. **(A)** Protein expression of the ER stress markers Bip, ATF6, p50-ATF6, IRE1α, p-IRE1α, PERK, and p-PERK. β-tubulin was used as an equal loading control (**Supplementary Figure 2**). **(B–H)** Representative densitometric analysis of western blots of these proteins. Data are means ± SEM. *n* = 3 (*p < 0.05; **p < 0.01; ***p < 0.001; WT, control; MM, missense mutation; SM, splicing mutation; FM, frameshift mutation).

The same ERS markers tested in iPSC were analyzed in fibroblasts. As showed in **Figures 4A, B**, we found a significant increase in Bip levels in WT fibroblast when compared with that of *COL4A5* mutant fibroblasts. Interestingly, the protein level of Bip in the *COL4A5* missense mutation fibroblasts was much higher when compared with that of the splicing mutation and frameshift mutation. As showed in **Figure 4** (ACDEFGH), the levels of p50-ATF6, IRE1α, p-IRE1α, PERK, and p-PERK in *COL4A5* mutant fibroblasts increased. Additionally, the levels of PERK in the missense mutation and frameshift mutation fibroblasts were much higher when compared with that of the splicing mutation fibroblasts, and the level of p50-ATF6 in the missense mutation was higher when compared with that of the splicing mutation and frameshift mutation fibroblasts. **Table 1** summarized the expression of ERS markers in fibroblasts and iPSCs containing different *COL4A5* genotypes.

**Table 1:** Expression of Each ERS Marker in Different Cell Types and Mutations.

	Bip Fibroblast/iPSC	ATF6 Fibroblast/iPSC	p50-ATF6 Fibroblast/iPSC	IRE1α Fibroblast/iPSC	p-IRE1α Fibroblast/iPSC	PERK Fibroblast/iPSC	p-PERK Fibroblast/iPSC
**Missense Mutation**	+^***^/*ND*	*ND*/*ND*	+^**^/*ND*	+^*^/*ND*	+^*^/*ND*	+^**^/*ND*	+^**^/*ND*
**Splicing Mutation**	+^**^/*ND*	*ND*/*ND*	+^*^/*ND*	+^**^/*ND*	+^*^/*ND*	+^***^/*ND*	+^*^/*ND*
**Frameshift Mutation**	+^**^/*ND*	*ND*/*ND*	+^**^/*ND*	+^**^/*ND*	+^*^/*ND*	+^*^/*ND*	+^**^/*ND*

+Upregulation of UPR markers.

ND,No significant difference to WT fibroblast and WT iPSC, respectively.

*Significant difference when compared with that of WT fibroblast and WT iPSC, respectively. (p < 0.05).

**Significant difference when compared with that of WT fibroblast and WT iPSC, respectively. (p < 0.01).

***Significant difference when compared with that of WT fibroblast and WT iPSC, respectively. (p < 0.001).

## Discussion

Most *COL4A5* mutations reside in the collagenous domain (Hertz, 2009). Thus, in our study, fibroblasts harboring different *COL4A5* gene mutations in the collagenous domain of the α5 (IV) chain were used to reprogram iPSCs, as described previously (Okita et al., 2011; Ling et al., 2019). The same hemizygous *COL4A5* gene mutations, identified in peripheral blood, were detected in Alport syndrome fibroblasts and iPSCs. The generated Alport syndrome iPSCs exhibited pluripotency markers Nanog and OCT4, had normal karyotypes, differentiated into three germ layers *in vitro* and expressed alkaline phosphatase. Alport syndrome specific iPSCs were generated successfully, which is consistent with previous studies (Kuebler et al., 2017; Ling et al., 2019). XLAS patient-derived iPSCs generated from *COL4A5* missense mutation and splicing mutation cells were reported previously (Chen et al., 2015; Kuebler et al., 2017; Haynes et al., 2018). Together with our results, we established *COL4A5* frameshift mutant iPSCs from fibroblasts for the first time, demonstrating that *COL4A5* mutation categories do not affect the reprogramming process.

Both dermal fibroblasts and podocytes are known to synthesize the α5 (IV) chain (Wang et al., 2005; Wang et al., 2012; Kruegel et al., 2013). However, it is difficult to obtain sufficient podocytes that originate from XLAS patients for further experiments. Thus, as an alternative, a small piece of a skin specimen from a XLAS patient is sufficient to culture skin fibroblasts for further studies (Wang et al., 2005). iPSCs are embryonic stem-cell-like pluripotent cells. The morphology, pluripotency markers epigenetic status, and gene expression profiles of iPSCs resemble embryonic stem cells (Takahashi and Yamanaka, 2006; Takahashi et al., 2007; Yu et al., 2007). iPSCs can self-renew *in vitro* and have the potential to differentiate to all cell types (Maherali et al., 2007; Wernig et al., 2007). Additionally, Zhao et al. reported that iPSCs are capable of generating fertile, live-born progeny by tetraploid complementation (Zhao et al., 2009). Thus, dermal fibroblast and iPSCs were chosen as tools for ERS activation in our study.

Under ERS conditions, unfolded proteins can dissociate the chaperone protein of ER-Bip from three major ER transducers, IRE1α, PERK, and ATF6, and this dissociation can activate these ER resident transducers and initiate UPR-mediated ERS (Cybulsky, 2017; Park et al., 2019). Therefore, we tested ATF6, IRE1α, and PERK, as well as their active forms, p50-ATF6, p-IRE1α, and p-PEKR, in WT and Alport syndrome fibroblasts. In this study, the upregulation of the three major ER transducers and their active forms indicated the activation of ERS in *COL4A5* mutant fibroblasts. A similar phenomenon has been reported (Wang et al., 2017; Nan et al., 2019).

To investigate whether mutant *COL4A5* can activate ER stress in stem cells, we detected molecules related to the ER stress pathway in different *COL4A5* mutant iPSCs. Noteworthy, no significant dysregulation of ER stress pathway markers (ATF6, IRE1α, PERK, and their active forms, p50-ATF6, p-IRE1α, and p-PEKR) was observed in the three *COL4A5* mutant iPSCs. Whether iPSCs have ERS defense-related mechanisms has not been studied. However, some studies showed that embryonic stem cells were resistant to many cellular stresses (Saretzki et al., 2004; Maynard et al., 2008; Saretzki et al., 2008), and iPSCs resemble embryonic stem cells. Therefore, we hypothesize that embryonic stem cells may have a special anti-ERS mechanism to maintain normal physiological functions and differentiation ability, and ensure that the genome remains stable after multiple passages.

We also detected significant activation of ERS in all three mutations in fibroblasts, and the missense variant caused stronger ERS in the Bip-ATF6 pathway. As ATF6 upregulates synthesis of ER chaperones to facilitate protein folding, the Bip-ATF6 pathway was called the “adaptive” UPR (Schroder and Kaufman, 2005; Cybulsky, 2017; Park et al., 2019). We postulate that activation of the ERS is associated with the primary sequence of α5 (IV) chain. Higher upregulation of the Bip-ATF6 pathway in the missense mutation may help cells to fold proteins more efficiently, form the triple-helix heterotrimer and consequently secrete the triple-helix heterotrimer to the extracellular matrix, whereas the Bip-ATF6 pathway may be less efficient when a truncated α5 (IV) chain is available. Therefore, patients with the missense mutant may present relatively mild phenotypes. However, characterization of the mechanisms responsible for the phenotypes requires further investigation in the future.

A strong correlation has been found between the *COL4A5* mutation type and the age developing end-stage renal disease in male patients (Kashtan et al., 2013; Nozu et al., 2019). It has been reported previously that phenotypic differences in *COL4A5* carriers can attribute to X chromosome inactivation (Shimizu et al., 2006; Wang et al., 2007; Rheault, 2012; Allred et al., 2015). Combined with the results of this study, we speculate that different *COL4A5* mutation types may activate different ERS signaling pathways, triggering different intracellular molecular reactions and leading to different clinical manifestations

There are limitations associated with this study. Obtaining sufficient kidney-derived cells that originate from XLAS patients is challenging and prohibits any kind of analysis. As an alternative, we analyzed ERS activation in fibroblasts and iPSCs, not kidney derived cells. As ERS activation differs in cell types, the results in this study may not be applicable to clinical applications. The UPR activation in kidney derived cells requires further investigation. Additionally, the sample size in this study is too small. Thus, further studies of more genotypes in ERS activation are required to further validate the observations presented.

In summary, we reprogrammed dermal fibroblasts of three different *COL4A5* mutation types (missense, splicing, and frameshift mutations) into iPSCs, and examined the effects of *COL4A5* mutations on ERS activation between undifferentiated stem cells and differentiated somatic cells for the first time. We confirmed that ERS activation varies between genotype and cell type, providing a theoretical basis for choosing the timing of intervention.

## Data Availability Statement

The raw data supporting the conclusions of this article will be made available by the authors, without undue reservation, to any qualified researcher.

## Ethics Statement

The studies involving human participants were reviewed and approved by The Ethics Committee of Peking University First Hospital. Written informed consent to participate in this study was provided by the participants' legal guardian/next of kin.

## Author Contributions

FW and JD conceived and designed the study. CW performed the experimental work and was assisted by SL, SX, KX, HX, and XW. CW performed the statistical analysis. CW wrote the first draft of the manuscript, and HD wrote a section of the manuscript. FW, LC, and JD edited the manuscript. All authors approved the final version of the manuscript.

## Funding

This study was supported by the National Nature Science Foundation of China (81070545 and 81570640), the National Key Research and Development Program of China (No. 2016YFC0901505), the registry study of rare diseases in children and Beijing key laboratory of molecular diagnosis and study on pediatric genetic diseases (BZ0317).

## Conflict of Interest

The authors declare that the research was conducted in the absence of any commercial or financial relationships that could be construed as potential conflicts of interest.

## Supplementary Material

Supplementary Figure 1 The full scan of the original gels of blots shown in **Figure 3**.

Supplementary Figure 2 The full scan of the original gels of blots shown in **Figure 4**.
