# Supplementary figures and images for: Endoplasmic Reticulum Stress Activation in Alport Syndrome Varies Between Genotype and Cell Type
Source: Front Genet. 2020 Feb 10;11:36. doi: 10.3389/fgene.2020.00036 (PMC7025644; doi:10.3389/fgene.2020.00036)

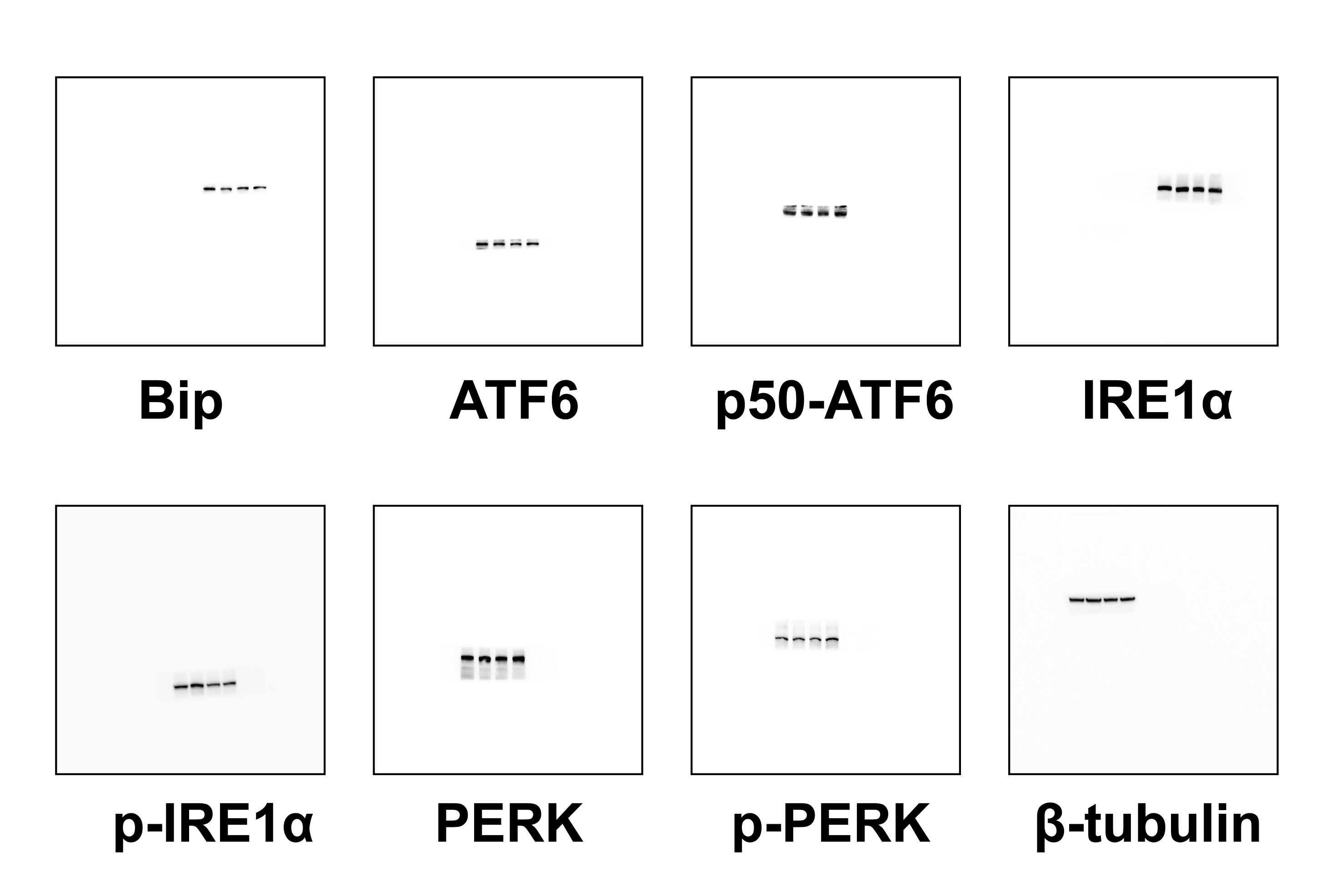

Supplement: Supplementary Figure 1 — The full scan of the original gels of blots shown in Figure 3 . [file Image_1.tif]

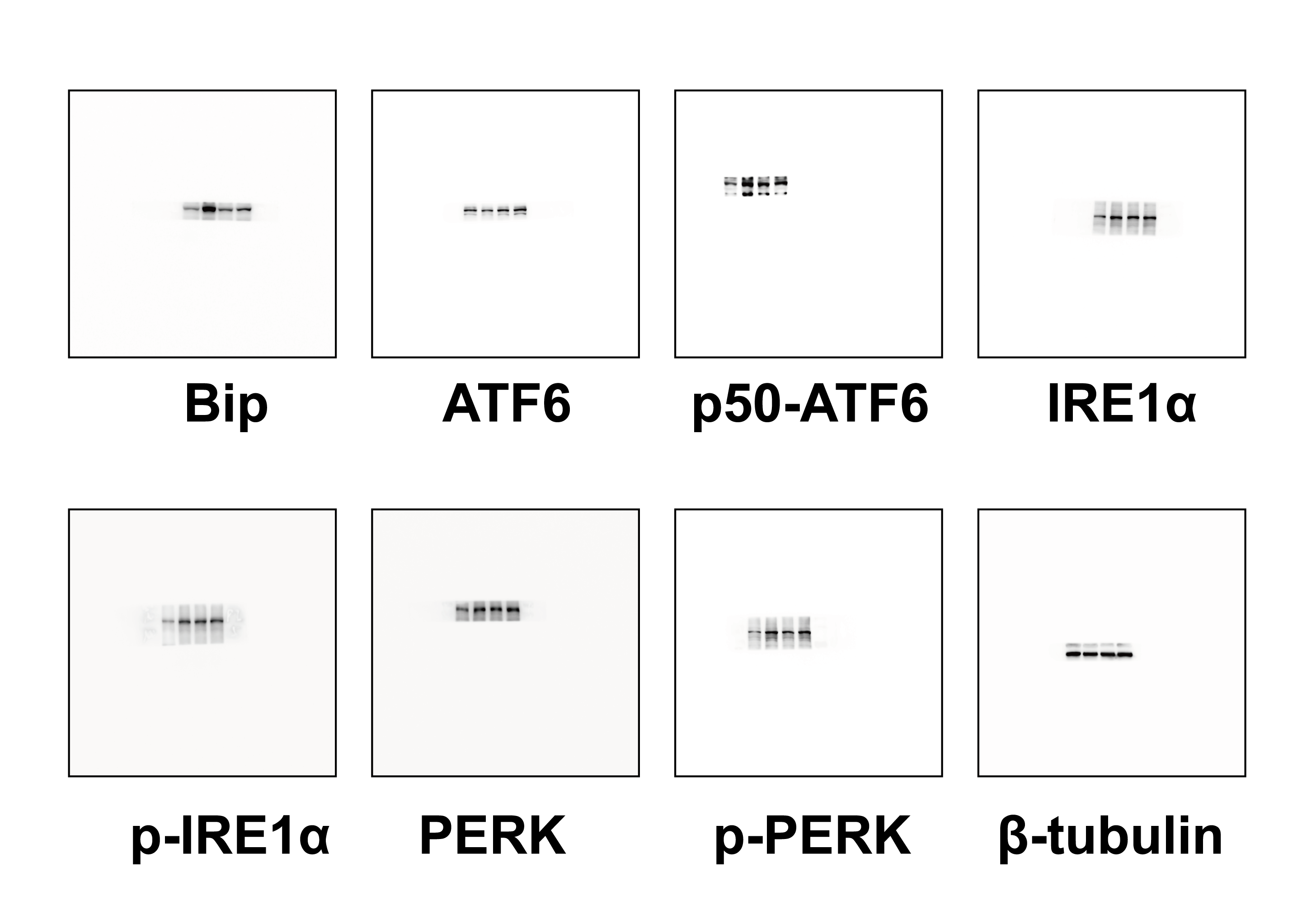

Supplement: Supplementary Figure 2 — The full scan of the original gels of blots shown in Figure 4 . [file Image_2.tif]
